# Programmable Design and Performance of Modular Magnetic Microswimmers

**DOI:** 10.1002/adma.202006237

**Published:** 2021-03-14

**Authors:** Christoph Pauer, Olivia du Roure, Julien Heuvingh, Tim Liedl, Joe Tavacoli

**Affiliations:** ^1^ Faculty of Physics and Center for NanoScience Ludwig‐Maximilians‐Universität Geschwister‐Scholl‐Platz 1 München 80539 Germany; ^2^ Physique et Mécanique des Milieux Hétérogènes CNRS ESPCI Paris Université PSL Sorbonne Université Université de Paris Paris F‐75005 France

**Keywords:** collective behavior, microactuators, microswimmers, self‐assembly

## Abstract

Synthetic biomimetic microswimmers are promising agents for in vivo healthcare and important frameworks to advance the understanding of locomotion strategies and collective motion at the microscopic scale. Nevertheless, constructing these devices with design flexibility and in large numbers remains a challenge. Here, a step toward meeting this challenge is taken by assembling such swimmers via the programmed shape and arrangement of superparamagnetic micromodules. The method's capacity for design flexibility is demonstrated through the assembly of a variety of swimmer architectures. On their actuation, strokes characterized by a balance of viscous and magnetic forces are found in all cases, but swimmers formed from a series of size‐graded triangular modules swim quicker than more traditional designs comprising a circular “head” and a slender tail. Linking performance to design, rules are extracted informing the construction of a second‐generation swimmer with a short tail and an elongated head optimized for speed. Its fast locomotion is attributed to a stroke that better breaks beating symmetry and an ability to beat fully with flex at high frequencies. Finally, production at scale is demonstrated through the assembly and swimming of a flock of the triangle‐based architectures to reveal four types of swimmer couplings.

## Graphical abstract

Magnetic micromodules of customized shape and position assemble to form flexible microswimmers. Through educated design, the swimming speed is optimized and the study of flocks of swimmers reveals new interactions between co‐moving magnetic beaters at the low Reynolds number limit.

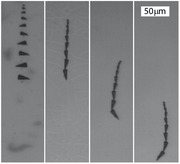

As set out in groundbreaking work by Purcell, locomotion at microscopic length‐scales rests on a swimming stroke that is non‐time symmetric.^[^ ^1^ ^]^ Purcell proposed a minimal example; a three‐rod system that generates propulsion through alternative rotation of its front and back appendages. Informed by Purcell and Nature itself, a number of artificial microswimmers have emerged over the last 20 years and are promising agents for targeted in vivo healthcare as well as important frameworks from which to advance the understanding of locomotion strategies at the microscopic scale.^[^ ^2^, ^3^, ^4^, ^5^, ^6^, ^7^, ^8^, ^9^, ^10^, ^11^, ^12^, ^13^, ^14^, ^15^, ^16^, ^17^, ^18^, ^19^, ^20^ ^]^ The swimming strategies employed by eukaryotic and prokaryotic cells that generate thrust by the sinusoidal‐like beating and the corkscrew rotation of flagellum, respectively, have been of particular inspiration to man‐made designs.^[^ ^4^, ^7^, ^13^, ^21^, ^22^, ^23^ ^]^ To mimic the former, and facilitate a non‐time‐symmetric swimming stroke, artificial microswimmers require jointed and/or inherently flexible frameworks that are typically actuated using external stimuli, for example, magnetic/electric fields, monochromatic light, or acoustic waves.^[^ ^2^, ^8^, ^9^, ^11^, ^22^, ^24^ ^]^ A seminal synthetic biomimetic microswimmer is a magnetoelastic one realized by Dreyfus et al., comprising a filament of monodisperse micrometer‐sized superparamagnetic beads connected together with DNA chains and tethered to a red blood cell.^[^ ^22^, ^25^ ^]^ Via coupling to a sinusoidal magnetic field, this swimmer moves through a paddle‐like beating of its flexible tail that initiates wave propagation predominately from its free to tethered end. Typically, the stroke of such swimmers and other magnetoelastic filaments depends on a balance of viscous, magnetic, and elastic forces. In cases where elastic forces are negligible, the balance requires only the former two forces, as quantified using the Mason number^[^ ^26^ ^]^

(1) $$\begin{array}{c} M_{a}=\zeta_{⊥}\mu_{0}\omega L^{2}/{\left(aB\chi \right)}^{2} \end{array}$$

Here, ζ_⊥_, μ_0_, ω, *L* are the perpendicular viscous coefficient, permeability of free space, angular frequency, and filament length, respectively, *a* is bead radius, *B* is magnetic field strength, and χ is magnetic volume susceptibility*. M*_a_ can be related to a magnetoviscous length scale, *l*_m_, as $M_{a}^{1/2}\approx \;L/l_{m}$, where the size of *l*_m_ with respect to *L* dictates the nature of the stroke in response to the time‐varied magnetic field. Specifically, the cases *l*_m_ ≫ *L* (magnetic forces ≫ viscous forces) and *l*_m_ ≪ *L* (viscous forces ≫ magnetic forces) are associated with rigid‐rod motion and tip wagging of magnetoelastic filaments, respectively, while at *l*_m_ ≈ *L* (viscous forces ≈ magnetic forces) their full rotation with flex is predicted.^[^ ^25^, ^26^, ^27^ ^]^

While many derivatives of this type of flexible biomimetic magnetic swimmer now exist, constructing them to specification and in large numbers remains a challenge, limiting their advancement toward application.^[^ ^2^, ^9^, ^21^, ^22^, ^23^, ^24^, ^25^, ^26^, ^27^, ^28^, ^29^ ^]^ Indeed, from an application perspective other types of synthetic micromotors have proved more fruitful such as rigid micromotors driven by surface‐mediated chemical reactions and/or magnetic fields and biohybrid designs that combine a man‐made element with a natural microswimmer.^[^ ^16^, ^17^, ^18^, ^28^, ^30^, ^31^, ^32^, ^33^, ^34^, ^35^ ^]^ Of the former class, rigid magnetically controllable biocompatible microcylinders propelled via reaction with gastric acid with drug release capabilities have been engineered as have magnetic rotators demonstrating long‐distance intravitreal propulsion within porcine eyes.^[^ ^18^, ^31^, ^34^ ^]^ Of the latter class, sperm cells coupled with magnetic elements have been constructed to allow their remote control and the targeted delivery of drugs or genetic material and, in general, the biohybrid approach is a promising one toward autonomous theranostics, microsurgery, and gene transfection.^[^ ^16^, ^17^, ^28^, ^33^ ^]^

Despite the relative lack of application progress, fully synthetic flexible microswimmers are in principal well suited for application as their fully engineered nature permits great scope to tune their performance for task. Those driven by magnetic fields are further suited for purpose because of the inherently bio compatible nature of the driving field and its capacity to be facilely tailored at low cost.^[^ ^29^, ^36^ ^]^ A particular bottleneck that effects these types of microswimmers, that more broadly impacts microrobotics, arises from the non‐triviality in engineering and then connecting microscale parts into configurations that yield precise actuations and the capacity to fabricate artificial microswimmers, and other microactuators, on the sub 100‐μm scale rapidly, robustly, and with design flexibility will greatly accelerate their progression to application and broaden their scope on arrival.^[^ ^15^, ^37^ ^]^

Herein, we outline a pathway to achieve these requirements using a new methodology to assemble modular, jointed magnetic microswimmers of pre‐programmable design. The modular units, which can have lengths as small as 2 µm, self‐assemble into microswimmers upon application of a homogenous magnetic field. By engineering these swimmers from discrete modules, we endow them with a flexibility essential for their swimming at the low Reynolds number limit.^[^ ^1^, ^11^ ^]^ Such flexibility is absent within single modules even at high aspect ratios prohibiting the fabrication of a swimmer from a single piece. To program swimmer architecture we use two handles: the shape of the modules and their spatial arrangement prior to assembly. The combination of these two variables with the applied field produces a magnetic landscape that rotates (magnetic torque) and/or translates (magnetic dipole‐dipole attraction/repulsions) the modules, thus assembling them into a structure of choice. The shape and angle dependency of magnetic torques and dipole–dipole interactions allow, in principle, the kinematic responses of our magnetic modules to be ab initio predicted.

We demonstrate the capacity for design flexibility by forming five distinct swimmers and reviewing their locomotion in the context of their form. This coupling brings understanding of the underlying physics and we extract design rules to optimize for swimming speed. Our parallel fabrication procedure allows us to study interactions between multiple beating synthetic biomimetic swimmers and we highlight four different types of couplings between our swimmers.

Control of the shape of our magnetic modules and their starting position is granted via a reported lithographical protocol to fabricate non‐spherical superparamagnetic particles and our fabrication and assembly approach is outlined in **Figure** **1**a.^[^ ^38^, ^39^, ^40^, ^41^ ^]^ The as produced particles—our modules—are composites of a densely and uniformly packed superparamagnetic colloid encased in a crosslinked network of the monomer ethoxylated trimethylolpropane triacrylate (ETPTA). All materials used have shown biocompatibility in ex vivo studies (Section S1, Supporting Information).^[^ ^41^ ^]^ Significantly, the homogenous packing of the magnetic colloid within the modules allows their magnetic easy axis to be geometrically defined, yielding a shape‐determined response to a magnetic field.^[^ ^38^, ^39^, ^40^ ^]^ In house measurements estimate the unitless magnetic volume susceptibility of our modules to be ≈1, that is, similar to commercial magnetic beads used for biological purifications (Section S2, Supporting Information).

**Figure 1:**
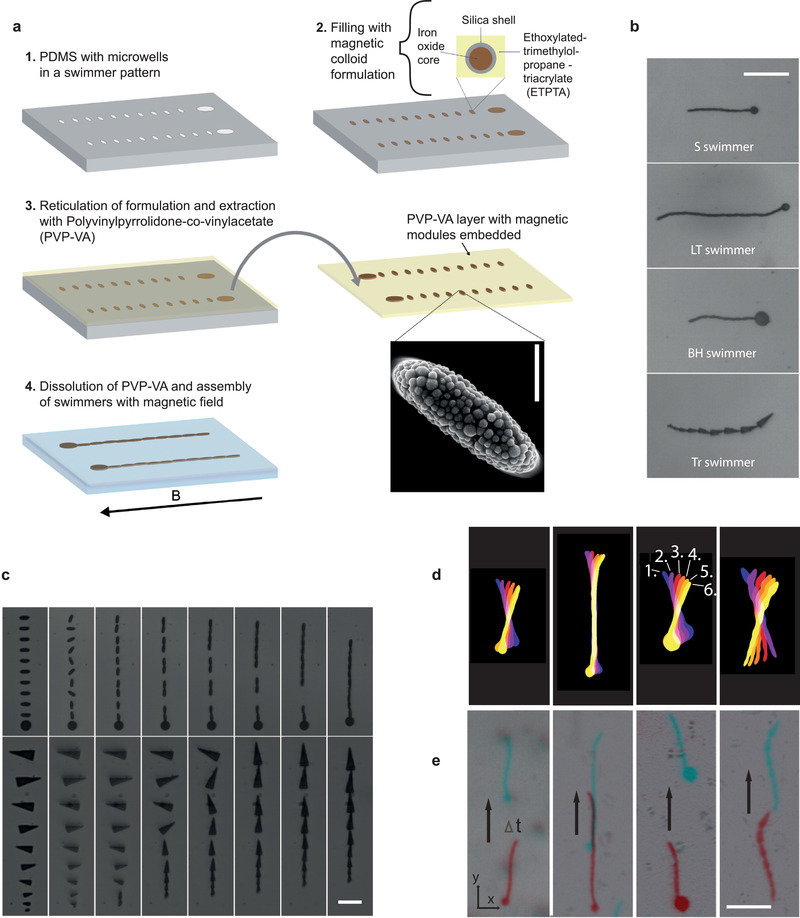
Fabrication, assembly, and swimming of microswimmers. a) Schematic of the fabrication and assembly of magnetic modules to microswimmers. The SEM image shows the packing of the colloid within modules. Scale bar: 2 µm. b) The swimmer designs. Scale bar: 50 µm. c) Assembly of an S swimmer (top) and a Tr Swimmer (bottom) under a magnetic field. The first frames are module release at *t*  =  0 s and timings thereafter (left to right) are 9.4, 9.75, 10.55, 11.20, 12.55, 14.55, 21.05 s (S swimmer); and 5.10, 6.20, 9.00, 10.30, 12.50, 15.30, 19.05 s (Tr swimmer). Scale bar: 20 µm. d,e) Time lapses demonstrating the half stroke (d) and locomotion direction (e) of the S, LT, BH, and Tr swimmers driven by a magnetic field alternating in the *x*–*y* plane (19 Hz). In (d) temporal evolution of the half stroke is indicated with the color scheme labelled 1–6, imaged by aliasing the 19 Hz beating at 20 fps capture. The aliasing gives the optical illusion of reverse swimming. Time intervals between the swimmers in (e) are 8.85 s (S, B, and LT swimmers) and 4.55 s (Tr swimmer). The arrows indicate travel direction and the red and blue colors indicate swimmer position before and after the time interval, respectively. Scale bar: 50 µm.

To highlight the design flexibility of our method we start by assembling four populations of distinct swimmer architectures: standard (S), long tail (LT), big head (BH), and triangle (Tr) (Figure 1b). The former three architectures (S, LT, and BH) are the same class of design, being head‐tail (HT) swimmers composed of a circular prism “head” connected to a series of smaller ellipsoidal‐prism “tail” units (long axis = 8 µm, short axis = 2 µm). These HT swimmers differ only through variation of head size (diameter = 8 µm (S, LT) or 16 µm (BH)) and number of tail units (10 (S, BH) or 20 (LT)). To facilitate the assembly of the HT architectures, the modules are linearly arranged with the major axis of individual tail modules offset 90° along the pattern. On application of a homogeneous magnetic field along the pattern's major axis (the *y*‐axis), the tail modules rotate, connecting tip‐to‐tip to form a tail that attaches on one end to the head module (Figure 1c, top). The Tr swimmer, to our knowledge a unique design, has a starting modular pattern of ten isosceles triangles, linearly arranged but with their major axes offset alternately at −80° and +80°. The leading two triangles have a long and short side of 20 and 8 µm respectively, thereafter the modules become systematically smaller by a factor 1–0.1*n* for each *n*th proceeding triangle (*n* = 1–8). The angular arrangement of the triangles induces their alternate counter clockwise/clockwise rotation which, in combination with their linear pattern, guides base‐to‐tip connections after their full rotation along the *y*‐directed field (Figure 1c, bottom). We emphasize that in both classes of swimmer design, rotation of the tail modules is a necessary assembly feature permitting the vast majority of swimmers to assemble to order (see Section S3, Supporting Information). Rotation of the module's major axis into the line of assembly allows the space between them to be rapidly reduced bidirectionally, thereby limiting their misalignment due to flow and/or Brownian motion before connection. Indeed, such misalignment was evident in trial designs with circular tail modules (i.e., modules holding no major axis) spaced at the minimum robust distance (≈3 µm) permitted by standard lithography, where module translation is the only means to close the distance between them (Section S4, Supporting Information). Furthermore, the programmed alternate counter‐clockwise clockwise rotation of the triangular modules of the Tr swimmer mitigates against undesirable tip‐to‐corner connections before their complete alignment to the external field.

To initiate locomotion of assembled swimmers, we combine a sinusoidally oscillating *x*‐directed magnetic field (peak modulus = 20 mT) to the static *y‐*field (25 mT) to produce a directionally time‐varied magnetic field in the *x*–*y* plane. This moving field induces beating of the swimmers as their dipole moments move to maintain alignment with it (Figure 1d). Strikingly, the vast majority of swimmers stay connected, both during swimming and after field cessation, despite the absence of polymer connections between their modules. Such polymer connections have been required in other microscale magnetic actuators to maintain structural integrity.^[^ ^22^, ^25^, ^26^, ^42^, ^43^ ^]^ We suggest the absence of charged groups on our modules permits their close contact (Section S5, Supporting Information, for SEM images of contacts between modules)—contact which is then stabilized by short ranged van der Waals interactions in addition to magnetic dipole–dipole attractions during swimming. We do, however, observe that a fraction of our swimmers breaks up and/or buckle at lower frequencies, particularly in the case of the BH architecture (Section S6, Supporting Information).

On swimming, the HT designs move tail first whereas the Tr swimmers translates with their largest triangle forward (Figure 1e). The tail‐first locomotion of our HT swimmers is reminiscent of an earlier magnetic swimmer, being a consequence of a field‐unresponsive head restricting the capacity of nearby tail units to follow the changing direction of the magnetic field.^[^ ^22^ ^]^ This situation produces bending waves that propagate from the untethered tip to the head producing a greater flow of liquid in the same direction and consequently an increased tail‐first propulsion force.^[^ ^25^ ^]^ Likewise, despite their magnetic nature, the head units of our HT swimmers seem able to “clamp” tail motion at the tethered end. Presumably this is because the head units lack a distinctive axis in the *x–y* plane due to their 2D isotropic shape and homogenous packing of magnetic colloids, which leaves them largely unaffected by the rotation of the field. Nevertheless, in all HT designs head‐end motion is not completely damped and some oscillation here is evident, albeit smaller than at the free end (**Figure** **2**a). The reverse situation is true of the Tr swimmers. These swimmers move head first and far more bending originates from the largest front triangle than the smaller ones at the rear. This breaking of beating symmetry takes place despite the fact that none of the Tr modules are clamped via attachment to a non‐rotating module, as in the HT swimmer situation.

**Figure 2:**
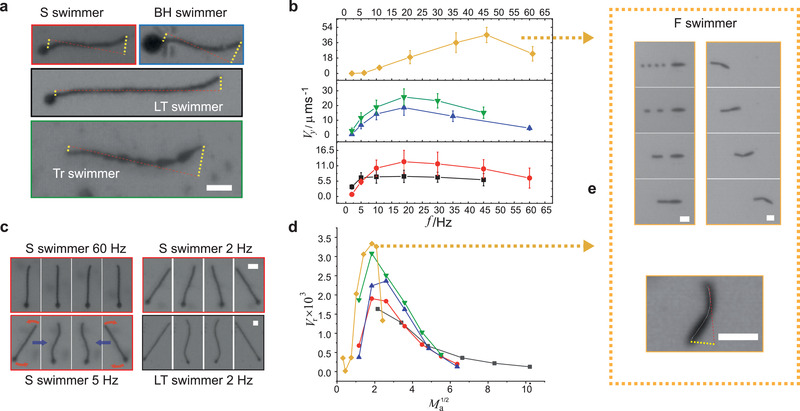
Swimming characteristics. a) Snapshots of S, BH, LT, and Tr swimmers during a stroke at 19 Hz with the amplitude of flex at both ends of the swimmers highlighted by dashed yellow lines. Scale bar: 20 µm. b) The absolute velocity, *V*_y_, of S (⚫), LT (◼), BH (▲), Tr (▼), and F (◆) swimmers with the frequency, *f*, of the oscillated magnetic field. The error bars are the standard deviation calculated from populations of swimmers. For each measurement between 5 and 35 swimmers were tracked. c) Snapshots of an S swimmer stroke and an LT swimmer stroke at 2 Hz. At 60 Hz, only the tips of the S swimmer actuate whereas at 5 and 2 Hz it rotates fully with flex and with a rigid‐rod‐like stroke, respectively. In contrast, the LT swimmer still displays flex at 2 Hz. The red arrows indicate the direction of actuation relative to the stroke extrema and the blue arrows point to the subsequent stroke confirmation. Scale bars: 20 µm. d) Plot of $M_{a}^{1/2}-V_{r}$ for S, LT, BH, and Tr swimmers. The graph key is the same as in (b). e) Left column: Fast swimmer assembly from its starting pattern of modules (left column). Timings from *t* = 0 (top panel) are 8.35 12.2, and 13.35 s. Right column: locomotion at 61 Hz showing full actuation of the F swimmer and it head first motion. Each image is 1 s apart. Bottom panel: image highlighting the flex along the length of the tail at 46 Hz. Scale bars are 20 µm.

As a consequence of our design control we are able to link our four distinct swimmer architectures to performance. Moving upward in sinusoidal field frequency from 2 to 60 Hz, we track the magnitude of the *y*‐velocity of our swimmers, *V*_*y*_, finding that in all cases it increases monotonically before peaking at ≈20 Hz and then decreasing more gradually (Figure 2b). Comparing the values of the peak velocity, *V*_*y*(peak)_, of our swimmers gives the following trend: Tr swimmer (≈26 µm s^−1^) > BH swimmer (≈18 µm s^−1^) > S swimmer (≈13 µm s^−1^) > LT swimmer (≈7 µm s^−1^). That the S swimmer is almost twice as fast as the LT swimmer is consistent with a *V*_*y*_ ∝ 1/*L* scaling theorized for other magnetoswimmers.^[^ ^22^, ^25^, ^26^ ^]^ This scaling arises from the independence of both the magnetoviscous and elastoviscous length scale, *l*_m_ and *l*_p_ respectively, from the length of the filament, *L* (the pertinent length scale, either *l*_m_ or *l*_p_, depends on the frequency, form, and makeup of the swimmer in question).^[^ ^22^, ^25^, ^26^ ^]^ As such, when *l*_m/p_ falls short of *L* a greater proportion of a shorter swimmer is providing thrust in comparison to a longer one. In accordance, we find that at 19 Hz the LT swimmer holds a larger rigid, non‐oscillating mid‐section—that presumably only offers extra load for transport—whereas the S and BH swimmers are almost fully actuated and flexed along their length (c.f. Figure 1d; Section S7, Supporting Information, for a full stroke). Using this argument that non‐actuating parts provide only load, it is unintuitive that the top velocity of the BH swimmer should exceed that off the S design; the former has a larger, heavier head that imparts increased drag. However, this bigger head further breaks the flexing symmetry of the tail to give a smaller stroke amplitude at the head end and a larger one at the free end with respect to the other HT designs. The increased tail‐first propulsion force that this stroke creates is seemingly more than compensatory for the BH swimmer's greater drag which is qualitatively consistent with simulations run on similar swimmers by Gauger et al.^[^ ^27^ ^]^ A similar line of argument can also explain the higher top velocity of the Tr design; its front‐end beats with a larger amplitude than the free ends of all HT designs. Furthermore, while a significant proportion of head‐end beating at the back works against free‐end beating in the HT designs, in the Tr case, back‐end beating is significantly more damped with flex disproportionally evolving from its largest lead triangle and extending almost to the body length of the swimmer. This greater amount of beating at one end should generate more net thrust and therefore higher swimming velocities. By correlating the lead angles (with respect to swimming direction) of the Tr and BH designs with progressive angles along their lengths, we find that the former exhibits more decorrelation over a larger proportion of its length during a stroke at 19 Hz—in support of the above observations (Section S8, Supporting Information). We suggest that the predominant front‐end beating of the Tr‐swimmer is a function of its graded modular size; larger triangles are able to impart greater torque and beating amplitudes that extend over greater lengths than smaller ones. In addition, the Tr‐swimmer should be more hydrodynamic than its HT counterparts. Its triangular shape (on a modular level) and its tapered width (on the swimmer body level) will decrease drag, both tangentially and parallel. Such features are a direct result of the shape, size, and arrangements of its modules, control of which is granted through our assembly method.

Despite their design differences, the swimmers display a similar range of stroke patterns. In fact, the S, BH, and Tr designs share the same frequency‐dependent nature of swimming (Figure 2c; Movie S1, Supporting Information). At 2 Hz these architectures fully rotate with the field, beating with a large rigid‐rod element (rigid‐rod‐like swimming). Between 5–30 Hz, for the S and Tr designs and 10–35 Hz for the BH architecture, the whole body of the swimmers remain actuated but their mid sections now lag the movement of their ends (full‐flex swimming). Within this frequency juncture, though the swimmers rotate fully the amplitude of their beating reduces with frequency. Finally, from 45 Hz onward, only the end parts of the swimmers actuate (end‐waggling swimming) with the length of these propulsive regions decreasing with frequency. In contrast, the LT design does not exhibit a rigid‐rod‐like stroke. Rather, at the lowest frequency (2 Hz), it swims with full flex (Figure 2c) before transforming into an end‐waggling stroke from 10 Hz upward. In other words, the S, BT, and Tr swimmers move from a regime where the magnetic force dominates the viscous force at the lowest frequency to a regime where the viscous forces dominate the magnetic forces at the highest. In the case of the LT swimmer, the magnetic and viscous force are already at equivalent magnitude at the lowest sampled frequency.

These different force regimes are fully distinguished by replotting Figure 2d in terms of reduced velocity, *V*_r_ = *V_y_*/*L*_s_ω, an indicator of swimmer efficiency, where we take *L*_s_ to be swimmer length, against an estimate of $M_{a}^{1/2}$ (Figure 2d; Section S9, Supporting Information). The use of $M_{a}$ is appropriate as we can neglect elastic forces due to the absence of polymer bonds between our modules. Now, the S, BH, and Tr designs range $M_{a}^{1/2}\approx$ 1 to ≈6.5 with a peak in *V*_r_ at $M_{a}^{1/2}$ ≈ 2 whereas the LT design extends from $M_{a}^{1/2}$ ≈ 2 to ≈10 with *V*_r_ reducing monotonically from *M*_a_^1/2^ ≈ 2. The peak in all $M_{a}^{1/2}-V_{r}$ curves corresponds to full‐flex swimming, the drop at higher frequencies to the end‐waggling stroke and its reduction at low frequencies—present for the S, BH, and Tr swimmers but absent in the LT design—to the rigid‐rod‐like stroke. This relationship between stroke character and *V*_r_ is consistent with numerical and theoretical studies on similar magnetic swimmers.^[^ ^25^, ^27^ ^]^ Full‐flex swimming gives high values of *V*_r_ as thrust is produced along the full length of the swimmer whereas end‐waggling and rigid‐rod‐like swimming produces lower values because of a reduction in thrust caused by only partial actuation and the greater reciprocal nature of the stroke, respectively.^[^ ^25^, ^27^ ^]^ The absence of the rigid‐rod‐like stroke for the LT swimmer is a consequence of its capacity to accommodate larger values of *l*_m_ before transitioning to full rotational swimming. Accordingly, for this design, full‐flex swimming is shifted from intermediate frequencies to the lowest frequencies of our experiments and end‐waggling manifests from intermediate frequencies onward—as reflected in its $M_{a}^{1/2}-V_{r}$ signature.

The above results and analysis offer clues to elevate swimming speed; designs that better break their beating symmetry and that have a full‐flex stroke at higher frequencies will swim faster. To meet these requirements, we design a fast swimmer (F swimmer) that consists of an elongated oval head module (24 µm by 6 µm) attached to a short tail composed of 4 circle modules (diameter 6 µm) (Figure 2e). Note, the small number of tail modules allows their circular shape because rotation is no longer required for robust assembly. Because the length of the F swimmer is considerably smaller than the other architectures (approximately twice as short as the S swimmer) it can now be fully actuated at the highest frequencies. Further, the back and forth rotation of the high aspect ratio head produces a greater beating amplitude at the head end than at the free end (Figure 2e, bottom panel; Movie S2, Supporting Information), to produce a stroke that appears to better break its beating symmetry than the proceeding 4 architectures examined.

A plot of *V*_*y*_ with frequency (Figure 2b, yellow diamonds) shows a velocity approaching 50 µm s^−1^ at 46 Hz, that is, significantly higher than previously realized, which progressively reduces at lower frequencies. This velocity is toward the top end of micromotor performance (Section S10, Supporting Information, for performance comparison of our swimmers to other leading micromotors). Plotting velocity results in terms of $M_{a}^{1/2}$ and *V*_r_ (Figure 2d, yellow diamonds) indicates our success in accessing full‐flex swimming at higher frequencies: the peak value of *V*_r_ corresponds to an $M_{a}^{1/2}$ at 36 Hz whereas for the initial set of designs the peak corresponded to 2 or 5 Hz. The rapid decline in *V*_r_ at smaller values reflects the earlier transformation to a rigid‐rod stroke due to the swimmer's shorter tail.

Beyond design flexibility, our assembly approach allows examination of the flock behavior of magnetoelastic swimmers. Understanding the interaction of co‐moving swimmers is of practical importance—a population of swimmers can deliver more therapeutic material than a single one alone, for instance, and for the majority of in vivo applications will have to act in unison.^[^ ^12^ ^]^ It is also of fundamental interest; out of equilibrium collective motion is a rapidly emerging field of Physics.^[^ ^12^, ^29^, ^44^ ^]^ However, so far, experimental and numerical works on cooperative effects of synthetic locomotors have largely focused on minimal designs with no degrees of motional freedom.^[^ ^12^ ^]^ These studies are important for extracting baseline physics of interest to both theorists and experimentalist but for obvious reasons can never probe the importance of specific features such as flagella length, beating frequency, stiffness, etc. that are suspected to influence large scale collective behavior in living systems.^[^ ^45^, ^46^, ^47^, ^48^ ^]^ Indeed, flexible biomimetic microswimmers are conspicuous through their absence, stymieing a decoupling of universal and case‐specific cooperative behavior as well as an understanding of systems that have potentially more benefit for application. This is not by choice; the preparation of precisely engineered biomimetic microswimmers is challenging, even more so when many are required to swim in unison.^[^ ^29^ ^]^ By employing self‐assembly to engineer joints between our swimmers we circumvent much of the taxing engineering typically required to make flexible synthetic microswimmers, thereby enabling their formation and locomotion in unison and with it a study of the collective interactions of flexible biomimetic microswimmers.

Starting from a rectangular array of Tr swimmers (≈50 swimmers) we witness their loose pattern formation in the early stages of locomotion; the rectangular lattice develops into a diamond‐shaped one (**Figure** **3**; Movie S3, Supporting Information). The evolution of this pattern is reminiscent of a spatial configuration generated from square arrays of magnetic discs in response to a homogenous magnetic field, to suggest the prominent role of magnetism rather than hydrodynamics.^[^ ^40^ ^]^

**Figure 3:**
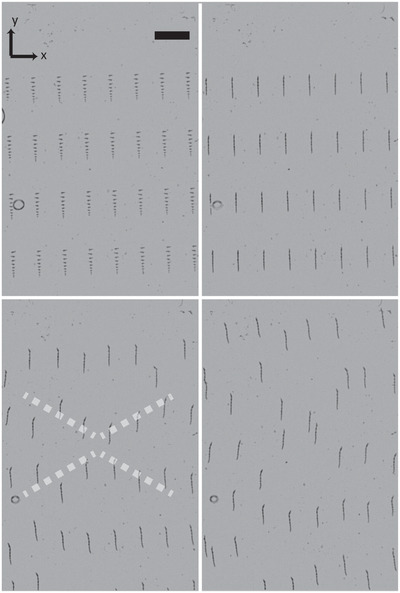
The assembly of a flock Tr swimmers and its evolution into a diamond‐shaped arrangement. The oscillating frequency is 39 Hz and the timings after assembly at *t* = 0 s are 18.1, 33, and 41.2 s. Scale bar is 200 µm.

After this initial patterning, the swimmers come together and interact predominately in pairs in four limiting cases: head‐to‐tail aggregation, side‐by‐side swimming, overtaking, and snap aggregation (**Figure** **4**; Movie S4, Supporting Information). The first example, head‐to‐tail aggregation, takes place when a rear swimmer catches up and connects with a slower one directly in front. During this process (Figure 4a, region 1), we record acceleration of the chasing swimmer and deceleration of the front swimmer as they approach within a distance of ≈15 µm. Attributed to both hydrodynamic and magnetic interactions, such acceleration effects have been anticipated in simulation work.^[^ ^49^ ^]^ On catchup (Figure 4a, region 2), connection does not take place immediately. First, a “metastable” situation is set up where the lead tip of the chaser and the back tip of the front swimmer are out of phase, causing repulsions as they move past each other close to contact. Eventually, the pair of swimmers permanently connect. In doing so, the back tip of the front swimmer is forced to beat in phase with the head of the chaser, to produce an arched beating stroke along the front half of the formed doublet. The shared velocity of the interacting, yet unconnected, pair of swimmers is ≈10% higher than that of the permanently connected doublet. The overtaking pairing (Figure 4b.) is self‐explanatory, a faster trailing swimmer moves past one in front. This process happens when the initially chasing swimmer approaches the one ahead with an *x*‐displacement. Now, rather than connecting with the front swimmer, the chaser overshoots to the side with both swimmers moving apart further along the *x*‐axis during the process. This *x*‐directed repulsion is consistent with unfavorable orthogonal/near orthogonal magnetic dipole–dipole interactions. Side‐by‐side swimming (Figure 4c) initiates from a similar starting condition than the overtaking coupling, but now the trailing swimmer does not pass the one in front. This coupling appears stable; the swimmers beat synchronously, do not assemble during the length of the experiments and maintain their relative positions and shared velocity. This interaction also yields an *x*‐component to swimming that is directed toward the *x*‐offset of the rear swimmer with respect to the one in front. Finally, snap aggregation (Figure 4d) initiates when a chasing swimmer is able to partially swim under/over one in front. On *y*‐overlap (due to *z*‐displacement), both swimmers separate along the *x*‐direction before rapidly snapping together and connecting over a portion of their lengths. As in the head‐to‐tail aggregation situation, acceleration and deacceleration of the back and front swimmer is evident on the former's approach, but in the case of cataclysmic aggregation a “metastable” state is not observed. Instead, aggregation takes place rapidly after some critical separation is reached. We present these flock interactions and swimmer couplings as first‐case scenarios enabled by our unique assembly protocol. Subsequent work, both experimental and theoretical, will provide further insight into the specific hydrodynamic and magnetic interactions that underlay them.

**Figure 4:**
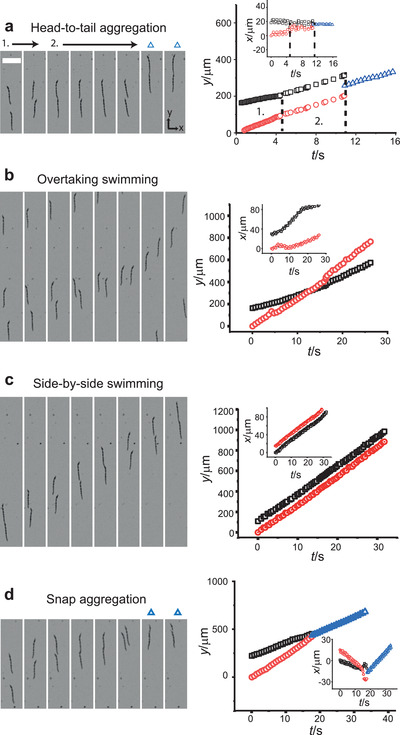
Swimming couplings. a–d) Image montages and trajectories of Tr swimmers during head‐to‐tail aggregation (a), overtaking (b), side‐by‐side (c), and snap aggregation (d) couplings. The montages are snapshots of the coupling interactions of the following timings (left to right): 1.00, 4.20, 4.25, 4.30, 4.35, 4.40, 12.90, and 13.30 s (head‐to‐tail aggregation); 10.45, 12.70, 15.15, 17.25, 19.90, 22.60, 28.25, and 36.55 s (overtaking); 3.00, 6.75, 10.50, 14.25, 18.00, 21.75, 25.50, and 29.25 s (side‐by‐side); 4.55, 10.80, 12.30, 13.05, 13.90, 16.95, 17.20, and 17.85 s (snap). Scale bar is 100 µm. The main graph panels show the *y*‐positions of lead swimmers (□), following swimmers (○), and aggregated states (Δ). The insets show swimmer *x*‐position. The partitions in graph (a) show the borders between the catchup process and when swimmers touch but do not aggregate.

In summary, we present a method to assemble flexible magnetic microswimmers that operate at the low Reynolds number limit. Assembly is programmed by the shape and relative position of the swimmers’ precursor magnetic modules. While maintaining tight control over design, this methodology frees us from time‐consuming approaches to engineer connections on the microscale and sidesteps the need for soft, flexible components that dependent on sophisticated processes for their development.^[^ ^2^, ^8^, ^9^ ^]^ Hence our non‐invasive approach to assembly marries adaptability of design with procedural ease to allow for the rapid design optimization of complex jointed engineered architectures, an asset that will facilitate designing for application in future iterations. We demonstrate this capacity by first engineering four bespoke swimming architectures and assessing their locomotion characteristics in the context of design. Using clues obtained from these experiments we design a further swimmer optimized for speed that contains a short tail enabling it to rotate with flex at high frequencies. Beyond design control of single swimmers, our assembly technique allows parallelization: many can be made in unison in preordained positions. Such parallelization reveals four distinct couplings of swimmers, offering new avenues for swimming optimization for application and study of the collective interactions of synthetic flexible biomimetic microswimmers for the first time. Looking ahead, we are confident that our assembly protocol can be extended to engineer a zoo of functional microrobotic devices: it remains a grand and interesting challenge to extract design rules that relate the vast combinations of starting arrangements and shapes of our modules to assembled design. Meeting this challenge will allow the production of microstructures of more complexity and functionality thereby extending application and understanding of locomotion on the microscale.

## Experimental Section

### Fabrication and Assembly of Magnetic Microswimmers

PDMS mold production: PDMS molds holding microwells of programmed arrangement and cross section were fabricated using standard soft‐lithographical techniques and templated by an SU8 resin as detailed in ref. ^[^ ^39^ ^]^.

Preparation of magnetic colloid precursor dispersion: A dispersion of magnetic colloidal particles with a silica shell (GE Healthcare, Serasil‐Mag, diameter = 400 nm) in the liquid monomer ethoxylated trimethylolpropane triacrylate (ETPTA, Sigma, *M*_n_ ≈ 428) was used as a precursor formulation to make the magnetic modules. To enhance the stability of the magnetic particles within ETPTA, the magnetic particles were first treated at room temperature at a concentration of 0.1 v/v% within a 5:1 methanol:ammonia_(aq)_ (10 wt%) solution with 0.5 v/v% 3‐(tremethoxysilyl)propyl methacrylate for two days. The now treated magnetic particles were then cleaned by five cycles of centrifugation and supernatant removal with methanol before finally transferring to ETPTA at 33 v/v% with 4 v/v% of the photoinitiator 2‐hydroxy‐2‐methyl‐1‐phenyl‐propan‐1‐one (Sigma) added to the final mixture.

### Magnetic Micromodule Fabrication and Extraction

PDMS microwells were filled with the magnetic colloidal dispersion in ETPTA by sliding a 20 µL droplet of it over the PDMS surface through gentle tilting of the mold. After filling, the dispersion was reticulated in the wells overnight under a 254 nm hand‐held UV lamp (NU4 KL, Benda Laborgeraete). To extract the now formed modular arrays, a 4 × 4 mm cross section of the mold was placed facedown onto a 25 µL drop of poly(1‐vinyl‐pyrrolidone‐*co*‐vinylacetate) (PVP‐VA), 70 wt% in isopropyl alcohol (Sigma), centered on a glass slide. The drop was bordered by ≈1 mm deep frame of PDMS of inner cross section proportions of 5 × 5 mm that forms a permanent seal with the glass slide. The slide was then left on hotplate at 200 °C for 20 s to rapidly evaporate off the isopropyl alcohol and melt the polymer. The mold was pressed into the polymer melt to ensure good contact and remove trapped bubbles and left to cool to room temperature. It was then gently peeled away to leave superparamagnetic arrays embedded in a flat layer of PVP‐VA.

### Assembly and Swimming of Microswimmers

For visualization of modular assembly and swimming, the glass slide holding the extracted modules was centered on a Zeiss Axiovert, 100M hal 100 microscope between a pair of NdFeB disc magnets (5 mm diameter, height 3 mm, superparamagnete) and magnetic coils (inner diameter 35mm, outer diameter 57 mm, height 15 mm 120 turns 0.85 mm Cu wire, Express Transformers) arranged orthogonally with a surface‐to‐surface separation of 75 mm and 30 mm, respectively. To ensure assembly of the swimmers, the long axis of their precursor modular pattern was aligned with the disc magnet pair. 50 µL ethanediol was then used to dissolve the PVP‐VA polymer and to release the magnetic modules. After assembly, a sinusoidal homogenous magnetic field was applied by the coil pair via a function generator (Agilent, 33220A) routed through an amplifier (P3000 Hafier Trans Nova) and a resistor (150 W, 3 Ω, ATE Electronics). Swimming as a function of frequency and assembly of the modules was captured using a microscope camera (USB 2.0 CMOS, Thorlabs) at 20 frames per second. The magnetic field strength produced at the center of the pair of disc magnets was 25 mT and the coils produced a maximum field strength modulus of 20 mT at their center.

## Conflict of Interest

The authors declare no conflict of interest.

## Supporting information

Supporting Information

Supplemental Movie 1

Supplemental Movie 2

Supplemental Movie 3

Supplemental Movie 4

## Data Availability

The data that support the findings of this study are available from the corresponding author upon reasonable request.
