# Supplementary material for: Programmable Design and Performance of Modular Magnetic Microswimmers
Source: Adv Mater. 2021 Mar 14;33(16):2006237. doi: 10.1002/adma.202006237 (PMC11469239; doi:10.1002/adma.202006237)
Supplement: Supplementary file 1 — Supporting Information [file ADMA-33-2006237-s002.pdf]

© 2021 Wiley-VCH GmbH

ADVANCED MATERIALS

Supporting Information

for *Adv. Mater.,* DOI: 10.1002/adma.202006237

Programmable Design and Performance of Modular Magnetic Microswimmers

*Christoph Pauer, Olivia du Roure, Julien Heuvingh, Tim Liedl, and Joe Tavacoli**

Supporting Information

S1: Ex-vivo compatibility

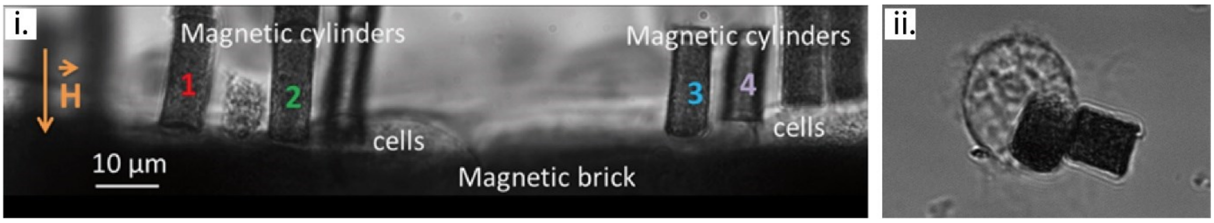

Figure S1: *Micrographs demonstrating ex-vivo biocompatibility of our magnetic micromodules. i.) Magnetic modules being used to probe the mechanical properties of epithelial cells themselves deposited on a magnetic substrate of the same materials as the modules. ii). The magnetic modules being ingested by a mouse dentritic cell. All biomaterials survived during the ~ 2 hour duration of experiments.*

Refer to reference 40 in main text for stability of actin to the magnetic modules (Sci Rep. 2017; 7: 15688. doi: *10.1038/s41598-017-15638-5).*

S2: Estimation of magnetic susceptibility

To estimate the magnetic susceptibility of our modules, we compare the rotation dynamics of 2 cuboid modules of the same form but of different magnetic composition. One of these modules is packed full of superparamagnetic particles purchased from GE Healthcare (*d*=400 nm) and the other with particles purchased from Ademtech (*d*=300 nm). The former composition is used in this study and the volume susceptibility of modules consisting of the latter particles has been previously measured. [S2.1]

To initiate cuboid rotation a homogenous magnetic field, aligned perpendicular to the cuboids long axis, was applied. The resultant rotation dynamics was fitted to:

$$\tan\theta\,(t) = \tan\,\theta_0\,\exp[-k(t-t_D)] \quad (1)$$

Where θ is the angle of the long axis of the cuboid with respect to the orientation of the applied magnetic field, θ_0 is the initial angle during the time interval before rotation, t_D, and k can be written as:

$$k = \frac{\chi^2}{(2+\chi)}\frac{\mu_0 g}{2\eta} D_{cub}^2 \left(\frac{H}{L_{cub}}\right)^2. \qquad (2)$$

Here χ is the magnetic volume susceptibility, μ_0 is the vacuum permeability, *g* is a slowly varying function of aspect ratio, D_{cub} and L_{cub} are cuboid length and width, respectively and *H* the magnetic field strength. [(S2.2)] χ can then be extracted from fitting *k* after first obtaining *g* from the Ademtech cuboids with known magnetic susceptibility. All other constants are known and the fitting gives a susceptibility, χ ~ 0.8, for the modules used in this study.

S2.1 Tavacoli, J. *et al.,* The fabrication and directed self-assembly of micron-sized superparamagnetic non-spherical particles. *Soft Matter* **9**, 9103–9110 (2013).

S2.2 Fresnais, J. *et al.,* Electrostatic Co-Assembly of Iron Oxide Nanoparticles and Polymers: Towards the Generation of Highly Persistent Superparamagnetic Nanorods. *Adv.Mater,* **20***,* 3877 (2008)

S3: Robustness of assembly process

Histogram distributions show that in the worst-case scenario, pertaining to the S swimmer, ~ 65% of the devices form correctly to length. In the LT swimmer case, 100% of designs appear to form correctly. We note that such differences do not stem from errors in the assembly process but rather from random defects in the PDMS molds presumably carried over from the production of their SU8 resin templates. These defects lead to shallow or blocked microwells and therefore on occasion missing module pieces.

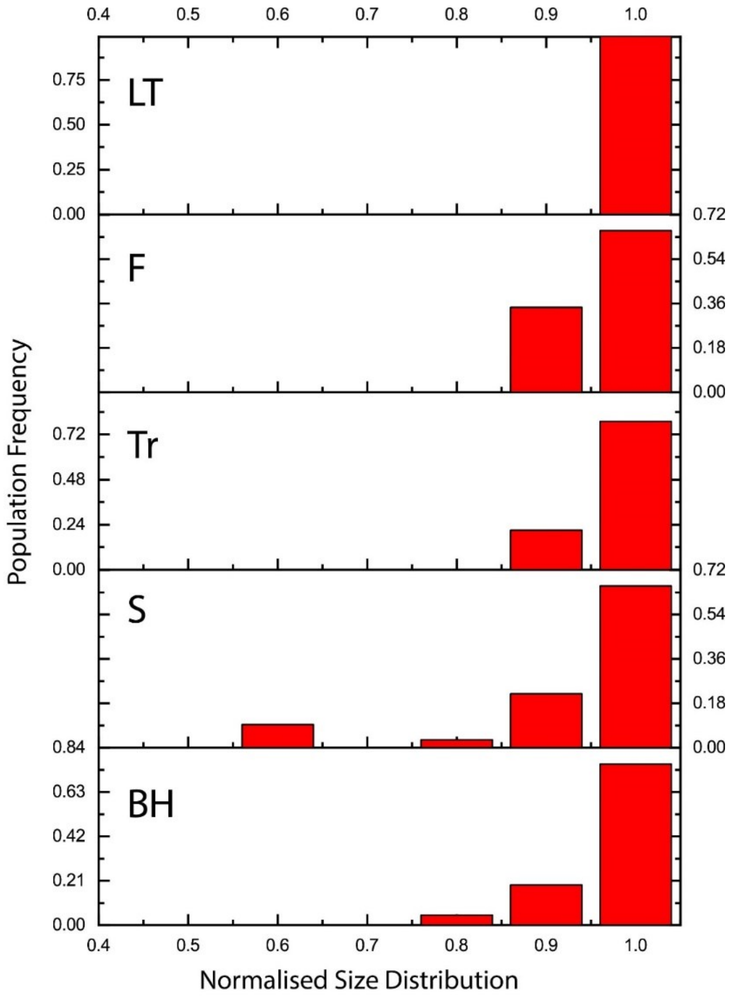

Figure S3: *Histogram of the normalized size distribution of the swimmers. Normalization took place by dividing the length of formed swimmers with the longest swimmer measured containing all of its modules. The number of counts for the BH, S, Tr F and LT swimmers are 28, 19, 30, 45, and 38, respectively.*

S4: Swimmer assembly with non-rotating modules

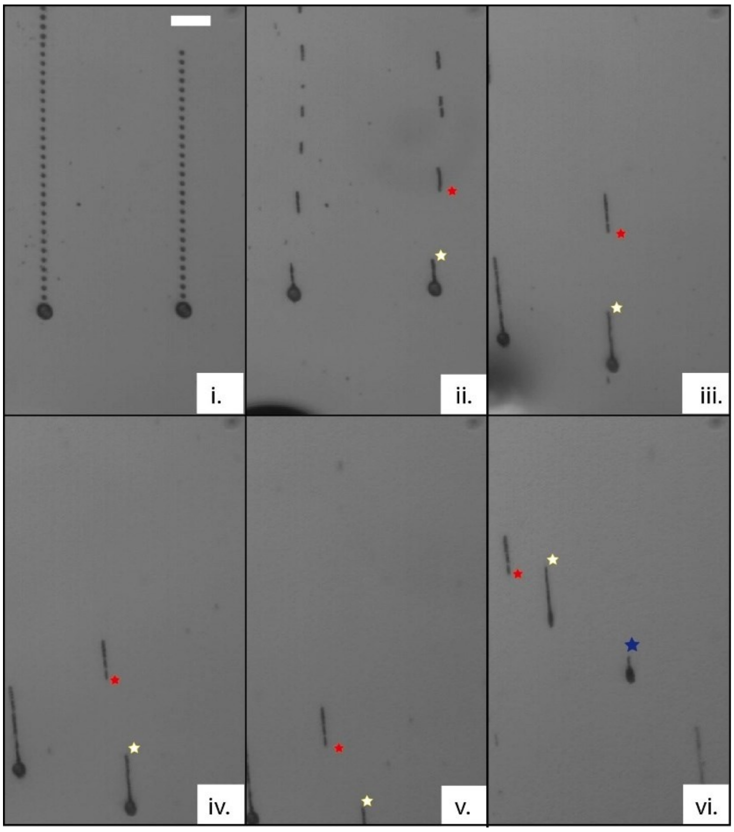

Figure S4: *Modular arrangement and design initially intended for assembly into a HT swimmer. The design consists of circular tail modules that do not rotate. This leads to a rapid build of space between sections of the tail which reduces their magnetic interactions and assembly rate, resulting in a tendency for flow to disrupt their alignment and therefore full swimmer formation. For clarity, the endpoints of the non-connected tail sections are highlighted with stars. In frame vi. the field of view is shifted to capture the incomplete swimmer. The blue star highlights a further incomplete swimmer pushed into the field of view by flow.*

The timings proceeding from i. to vi., after initiation of assembly, are 0 s, 33.2 s, 222.4 s, 307.8 s, 478.8 s and 517.2 s. A 25 mT homogenous magnetic field was applied and the scale bar is 20 microns.

S5: Swimmer connections

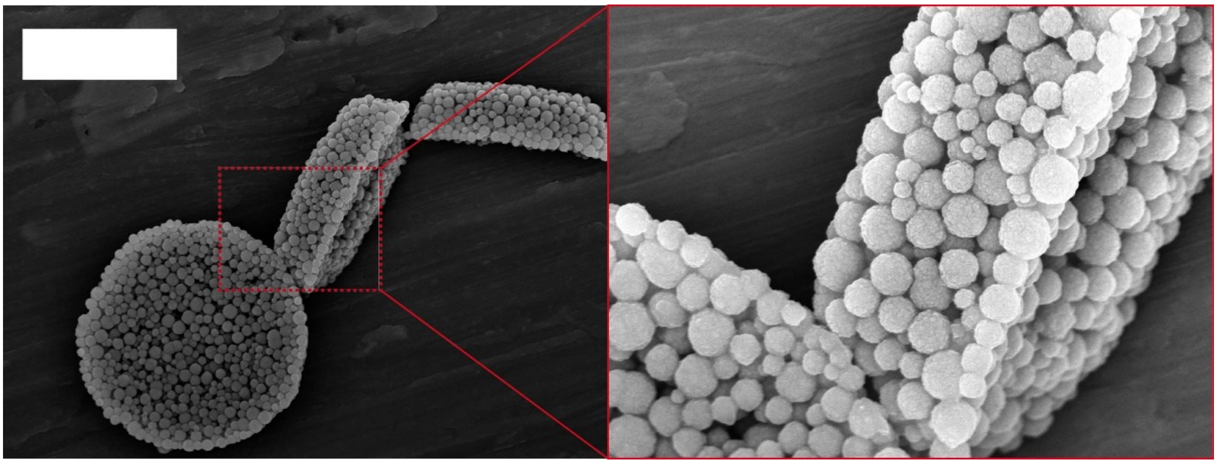

***Figure S5**: SEM micrographs showing direct connections between the magnetic modules. The scale bar is 5 microns.*

S6: Swimmer breakdown

A proportion of the magnetic swimmers break down during swimming - especially at the lower end of frequencies where the beating amplitude is higher and flex significant. This is particularly true of the BH swimmer - possibly as this architecture (of the HT designs) imparts the most flex during a stroke and its tail modules are more attracted to the larger head module.

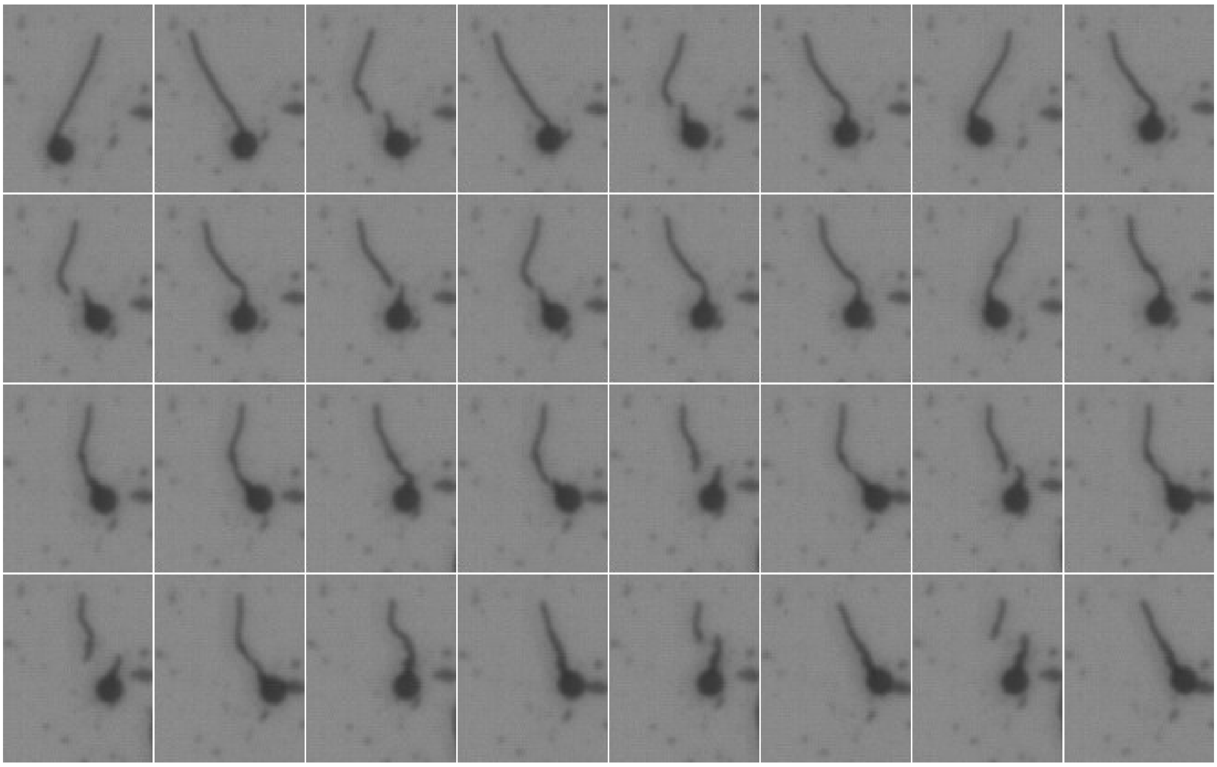

Figure S6. *Break down of a HT swimmer at 10 Hz. The figure proceeds left to right then down and each frame is 0.15 seconds apart.*

S7: Swimming at 19Hz

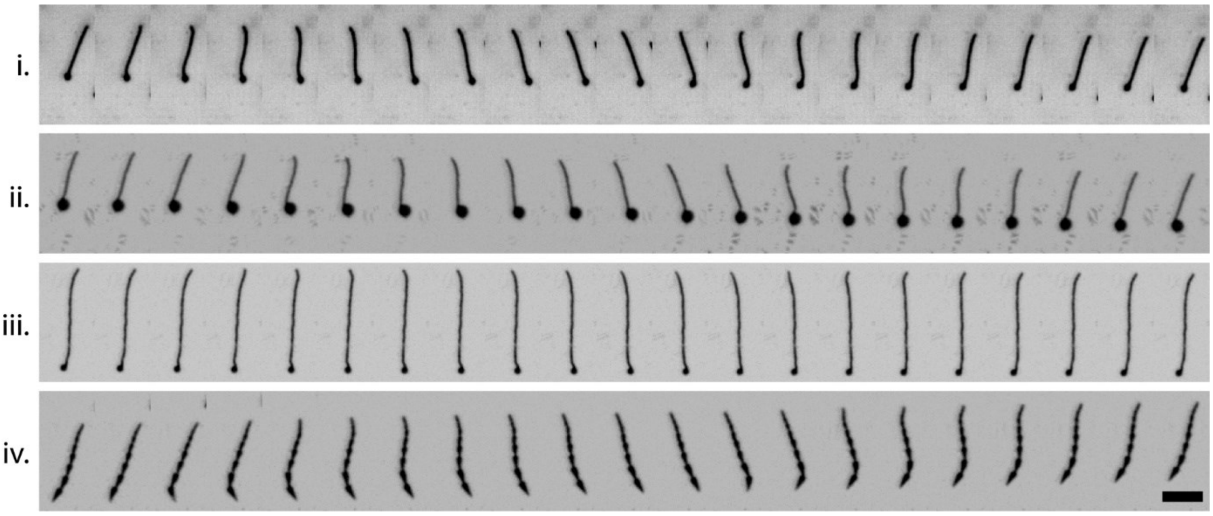

Figure S7. *Full strokes of the S (i.), BH (ii.), LT (iii.) and Tr (iv.) swimmers at 19 Hz. Full actuation of the S, BH and Tr swimmers is evident while the LT swimmer performs only end wagging. The stokes were imaged by capturing a 19 Hz beating pattern at 20 fps stroke to produce aliasing. The aliasing produces the optical illusion of swimming in reverse in these captions. Scale bar is 50 microns.*

S8: Angle correlations of Tr and BH swimmers during a half stroke

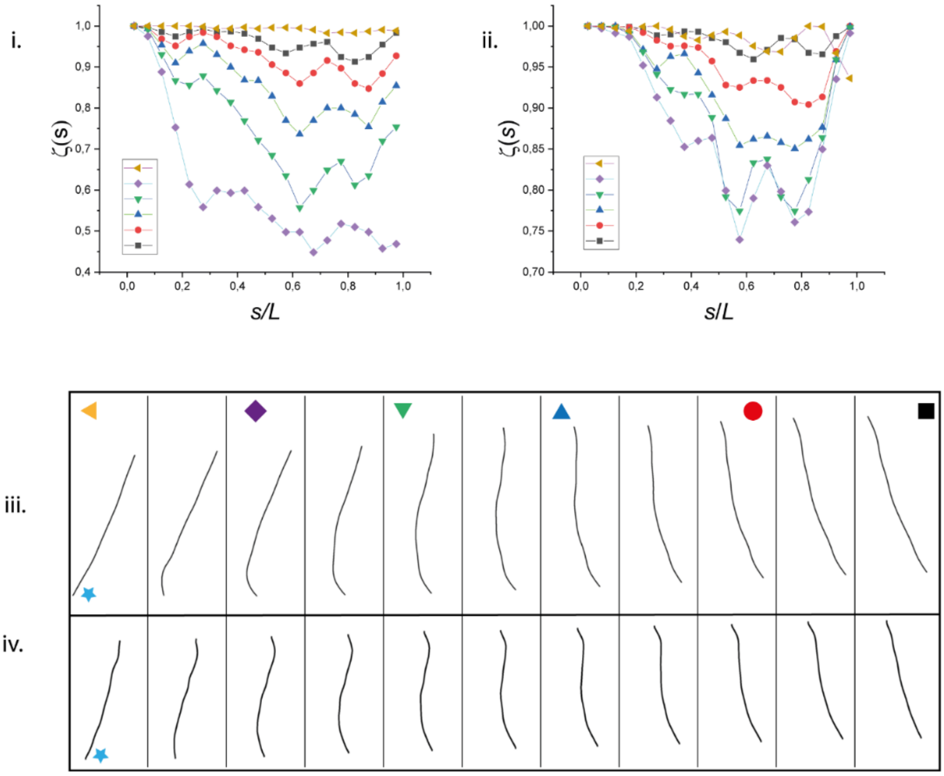

Figure S8. *Angular-pair correlation function, $\zeta(s)$ of the Tr swimmer (i.) and the BH swimmer (ii.) over half a stroke. $\zeta = cos(\theta_1 - \theta_s)$ where θ_1 is the angle at the front end of the swimmer during a stroke and θ_s is the corresponding angle a distance, s, along its tail length, L.*

The plots in graphs i. and ii. correspond to skeletons of the swimmer configurations in montages iii. and iv., respectively, as highlighted with the colored symbols. The blue stars in the starting frame in iii. and iv. indicate the large-triangle end and the free end of the Tr swimmer and BH swimmer, respectively. The half stroke was imaged by capturing a 19 Hz beating pattern at 20 fps stroke to produce aliasing. The aliasing produces the optical illusion of swimming in reverse in these captions.

The lower values of $\zeta(s)$ reached by Tr swimmer indicate it has a larger beating amplitude at its front triangle than the HT swimmer obtains at its free end. In addition, the decorrelation of the Tr swimmer can extend across its length – especially evident at the start of the stroke (purple diamonds in figure) – showing beating is one sided and wave propagation one way. In contrast, the BH swimmer shows more two-sided beating across its stroke as reflected by a return to full correlation commencing from s/L>0.8.

S9: Estimating $M_a^{1/2}$

To estimate $M_a^{1/2}=(\zeta_\perp\mu_0\omega)^{1/2}L/aB\chi$ for the HT swimmers we use the known values of μ_0, ω, L, B, χ, take a to be the half thickness of our module pieces and estimate $\zeta_\perp$ *using* $\zeta_\perp = \frac{2\pi\eta}{log(L/a)-1/2}$, the perpendicular drag coefficient for a prolate spheroid.(S9.1) This has been shown to be close to the drag coefficient originating from a chain of spheres of the same aspect ratio.(S9.2) Our case is still distinct due to the 2D nature of our modules but in the case of the HT swimmers: module depth ~ module width ~ 2 μm and scaling with V_r-$M_a^{1/2}$ collapses their V_y-f data points, suggesting a balance of viscous and magnetic forces dictates their stroke. TheTr and F swimmers incorporate different modular geometries than the HT designs which will, at the very least, change the form of their required perpendicular drag coefficient and also creates ambiguity of what value of a to use. In the absence of this information, and to aid discussion, we arbitrarily align their peak V_r values to the HT designs' peak at $M_a^{1/2}$ ~ 2. This does not change the form of their M_a-V_r signatures which is the key information used to understand the balance of elastic and magnetic forces acting on the swimmers, the information leveraged to optimize swimming velocity.

References

S9.1 Cox, R. G., The motion of long slender bodies in a viscous fluid. Part I. General Theory. *Journal of Fluid Mechanics* **44**, 791-810 (1970)

S9.2 Zahn, K., Lenke, R., and Maret, G., Friction coefficient of rod-like chains of spheres at very low Reynolds numbers. Part I. Experiment, *Journal de Physique II* **4,** 555-560 (1994) and Meunier, A., Friction coefficient of rod-like chains of spheres at very low Reynolds numbers. Part II. Numerical simulation, *Journal de Physique II* **4**, 561-564 (1994)

S10: Performance comparison of our swimmers to other leading micromotors

Ref.	Ls/µm	Micromotor Type	Locomotion Source	Directed locomotion (Yes/No)	V/µms-1	New fabrication concept?	Materials	POC application demonstrated?	Bio compatibilty	Flocks (Y/N)
This work	~100	Synthetic Biomimetic. Self-assembled joints.	Magnetic field (actuation)	Y, magnetic field	~50	Y	Silica, Iron Oxide, ETPTA	N	Stable to cells and proteins (ex vivo)	Y
S10.1	~100	Synthetic Biomimetic Pre-engineered joints	Magnetic field (actuation)	Y, magnetic field	~30	N	Nickel photoresist	N	Not demonstrated	N
S10.2	~5	Synthetic Pre-engineered joints	magnetic field (actuation)	Y, magnetic field	~50	N	Gold Nickel Silver	N	Not demonstrated	N
S10.3	~100	Bio. Hybrid	Magnetic field rotation)	Y, magnetic field	~50	N	Sperm Cell Iron Titanium Photoresis*	Y, in vitro directed drug delivery	Not demonstrated	N
S10.4	~100	Bio. Hybrid	Magnetic field (actuation)	Y, magnetic field	~50	Y	Sperm Cell Iron Oxide	Y, drug capture and release	Stable to cells (ex vivo)	N
S10.5	~20	Janus Particle	One sided Chemical reaction	N	_	N	Zinc Gelatin PEDOT Enteric polymer	Y, drug capture and triggered release (in vivo)	Y (in vivo)	Y
S10.6	~15.5	Synthetic Pre-engineered joints	magnetic field (actuation)	Y, magnetic field	Up to 15	Y	PPy Nickel PAH PSS	N	Not demonstrated	N
S10.7	~0.34	Nanobots	Enzymatic Catalysis	N	-	N	Silica MSNP APTES Urease	Y, drug capture and release (in vitro)	Y (in vitro)	Y
S10.8	~0.06 5	Janus Particle	One sided chemical reaction	N	-	N	MSNP Platinum	Y, cargo capture and release (in vitro)	Not demonstrated	Y
S10.9	1.4-2.6	Janus Particle	One sided chemical reaction	N	-	N	PLL Urease	Y, drug capture and release	Y (in vitro)	Y
S10.10	~50	Metallic Microtubes	Chemical reaction+ external magnetic field(for Fe)	Y	~275	N	Ti/Fe/Pt	Y, cargo transport	Not demonstrated	Y
S10.11	1.5-2.0	Microtubes	Acoustically driven	N	~50 (in water) ~10 (in blood)	N	Gold RBC PL	Y, detoxification	Y (in situ)	Y
S10.12	~11	Janus Nanotree	Phototaxis	Y, light controlled navigation	-	N	Silicon TiO2	N	Not demonstrated	N

L_s: Swimmer Length

V: Swimmer Velocity

MSNP: mesoporous silica nanoparticle

RBC: red blood cell membrane

PL: platelet membrane

References

S10.1 *Magnetically Driven Undulatory Microswimmers, Integrating Multiple Rigid Segments*, Small 2019, 15, 1901197, https://doi.org/10.1002/smll.201901197

S10.2 *Highly Efficient Freestyle Magnetic Nanoswimmer, Nano Lett.* 2017, 17, 8, 5092–5098, https://doi.org/10.1021/acs.nanolett.7b02383

S10.3 *Sperm-Hybrid Micromotor for Targeted Drug Delivery*, ACS Nano 2018, 12, 327−337 https://doi.org/10.1021/acsnano.7b06398

S10.4 *IRONSperm: Sperm-templated soft magnetic microrobots, Science Advances* 2020: 6, 28, eaba5855 https://doi.org/10.1126/sciadv.aba5855

S10.5 *Multicompartment Tubular Micromotors Toward Enhanced Localized Active Delivery*, *Adv. Mater.* 2020, *32*, 2000091 https://doi.org/10.1002/adma.202000091

S10.6 *Undulatory Locomotion of Magnetic Multilink Nanoswimmers*, *Nano Lett.* 2015, 15, 7, 4829–4833, https://doi.org/10.1021/acs.nanolett.5b01981

S10.7 *Enzyme‐Powered Nanobots Enhance Anticancer Drug Delivery*, S., *Adv. Funct. Mater.* 2018, 28, 1705086. https://doi.org/10.1002/adfm.201705086

S10.8 *J. Am. Chem. Soc. 2015, 137, 15, 4976–4979,* https://doi.org/10.1021/jacs.5b02700

S10.9 *Enzyme-powered Janus platelet cell robots for active and targeted drug delivery,* Tang et al., Sci. Robot. 5, eaba6137 (2020)

S10.10 *Magnetic Control of Tubular Catalytic Microbots for the Transport, Assembly, and Delivery of Micro-objects,* Adv. Funct. Mater. 2010, 20, 2430–2435, DOI: 10.1002/adfm.200902376

S10.11 *Hybrid biomembrane–functionalized nanorobots for concurrent removal of pathogenic bacteria and toxins,* Esteban-Fernández de Ávila et al., Sci. Robot. 3, eaat0485 (2018)

S10.12 *Programmable artificial phototactic microswimmer, Nature Nanotech 11, 1087–1092 (2016). https://doi.org/10.1038/nnano.2016.187*

Movies

Movie 1: S swimmer stroke with frequency. Scale bar is 50 μm.

Movie 2: F-Swimmers at 36 Hz. Scale bar is 50 μm.

Movie 3: Population swimming and pattern formation (scale bar 200 μm)

Movie 4: Pair Swimming (i.=head-to-tail aggregation, ii.=side-by-side swimming, iii.=overtaking and iv. = snap aggregation, scale bar 100 μm)

All movies are played at 60 fps and were recorded at 20 fps.
